# Different treatment modalities for advanced laryngeal cancer: improvement of oncological outcomes after tumor board implementation

**DOI:** 10.1016/j.clinsp.2025.100836

**Published:** 2025-11-21

**Authors:** Mariana Gonçalves Rodrigues, Daniel Marin Ramos, Leandro Luongo de Matos, Chin Shien Lin, Jorge Tomio Takahashi, Tiago Wanderley Diniz Chamel, Luiz Paulo Kowalski

**Affiliations:** a Head and Neck Surgery, Department of Surgery, Faculdade de Medicina da Universidade de São Paulo, São Paulo, SP, Brazil; b Department of Radiology, Faculdade de Medicina da Universidade de São Paulo, São Paulo, São Paulo, SP, Brazil

**Keywords:** Laryngeal cancer, Total laryngectomy, Chemoradiotherapy, Tumor board, Oncological outcomes

## Abstract

**Introduction:**

Laryngeal Squamous Cell Carcinoma (LSCC) has several treatment modalities available. Contrary to what has been observed in other cancers, the survival rates have worsened in recent years.

**Methods:**

Retrospective cohort study evaluating patients with T3 and T4a LSCC divided into two groups: those who were submitted to Total Laryngectomy (TL) or to Chemoradiation (CRT) or radiotherapy alone with curative intent between 2009 and 2019.

**Results:**

Three hundred and twenty patients were considered eligible to participate in the study, of which 166 (51.87%) submitted to TL and 154 (48.12%) to CRT. Overall survival in the surgical group was higher compared to the CRT group only between 2009 and 2013. In 2014, a multidisciplinary meeting was organized at the institution where the study took place, and both treatment modalities reached similar oncological outcomes.

**Conclusion:**

Multidisciplinary discussion among experienced professionals has a positive impact on oncological outcomes.

## Highlights

• Laryngeal cancer can be treated with total laryngectomy or chemoradiation therapy.
• Total laryngectomy is the main treatment for patients with T4a stage tumors.
• A multidisciplinary discussion has a positive impact on the oncological outcomes.

## Introduction

Laryngeal Squamous Cell Carcinoma (LSCC) is one of the most frequently diagnosed head and neck neoplasms. Several treatment modalities are used with the aim of achieving cure, and whenever possible, functional preservation. For tumors diagnosed at early stages, Radiotherapy (RT) and endoscopic partial laryngectomies are the main therapeutic options^1^^,^^2^. In cases of advanced cancer, Total Laryngectomy (TL) has long been considered the gold-standard treatment for advanced LSCC. However, in 1991, a prospective randomized study showed the non-inferiority of survival rates of advanced LSCC patients treated with neoadjuvant chemotherapy, followed by radiotherapy in relation to a group surgically treated^3^. In the following decades, there has been a reduction in the number of total laryngectomies performed, and a concomitant increase in non-operative, or “conservative” treatments of the larynx^4^, ^5^, ^6^. However, contrary to what has been observed in other cancers, the survival rates of patients with LSCC have worsened in recent years^7^^,^^8^.

Reference oncological services often have multidisciplinary meetings, or “tumor boards”, for the discussion of complex cases and shared decisions^9^^,^^10^. Cases of patients with advanced LSCC are usually discussed in such meetings, not only because the best treatment modality is not a consensus in the literature, but also because the particularities of each individual must be taken into account. Pulmonary function, age, comorbidities, education, personal desires, and even the profession of a patient can influence the choice of treatment modality^11^, ^12^, ^13^.

The aim of this study was to compare the oncological outcomes of patients with advanced LSCC undergoing surgical treatment or radiotherapy with or without chemotherapy, at a single institution, before and after the implementation of the institution’s tumor board. Secondly, the authors studied whether the implementation of a tumor board impacts survival

## Patients and methods

This was a retrospective cohort study, conducted at the Instituto do Câncer do Estado de São Paulo do Hospital das Clínicas da Universidade de São Paulo (ICESP HCFMUSP), the greatest public Cancer Center of Latin America. The study was also approved by the Institutional’s Review Board (CAAE: 38,792,420.1.0000.0068).

Initially, all patients registered at the Hospital’s system under ICD-10 C32 (malignant neoplasm of larynx) were identified. All 1631 medical records of patients with LSCC admitted from 2009 to 2019 were reviewed. Five hundred and eighty-one patients whose pretreatment primary tumor staging was T3 or T4a (by the eighth edition of the AJCC TNM system) and who were previously untreated were selected.

Patients with distant metastases, those whose treatment objective was palliative, and patients with recurrent, previous, or secondary primary malignancies were excluded. All surgical patients underwent TL with or without neck dissection and postoperative adjuvant therapy. Patients undergoing non-operative treatment received chemotherapy (cisplatin) and radiotherapy or radiotherapy alone, with at least a linear particle accelerator (total dose between 6600 and 7000 cGy. Intensity Modulated Radiotherapy [IMRT] ‒ or conformational Radiotherapy-3D RT ‒ were used during the study period). Patients who did not complete Chemoradiation Therapy (CRT) due to complications or death during treatment were not excluded from the study in an intention-to-treat model. Likewise, patients with postoperative complications or surgery-related death were also retained in the study.

Participants were organized into two groups: those who underwent TL and those who received curative intent CRT. Both groups were compared by lymph node, sex, age, Body Mass Index (BMI), the Eastern Cooperative Oncology Group (ECOG) score for functional status of cancer patients, and the Karnofsky Performance and Status Scale (KPS) to evaluate the homogeneity or not between them. All pre-treatment neck CT-scans were reviewed by two head and neck specialist radiologists (J.T. and T.C.), to exclude possible patients with inoperability criteria from both groups. The inoperability criteria investigated in the CT-scans were invasion of the prevertebral fascia, invasion of the deep muscles of the neck, invasion of the internal carotid artery, invasion of the common carotid artery, invasion greater than 2 cm from the base of the tongue, extension above the soft palate, and extension below the level of the cricopharyngeal muscle. Patients with any of those characteristics were also excluded.

It was only in 2013 that cases of advanced LSCC began to be discussed by the institutional head and neck tumor board. The multidisciplinary meeting between head and neck surgeons, radiologists, clinical oncologists, pathologists, radiotherapists, and all multidisciplinary team takes place weekly, when the group discusses more complex cases to improve patient’s care. Prior to 2014, new institutional patients with LSCC could be referred directly to both the clinical oncology and radiotherapy teams or to the head and neck surgery team. The treatment was often defined at the first medical consultation, without a multidisciplinary discussion. Based on that, participants were subdivided again, according to the period in which their main treatment was started: from 2009 to 2013, before the tumor board, and between 2014 and 2019, after the tumor board. Survival status one year after the main treatment conclusion (surgery date or last CRT Day) was accessed.

### Statistical analysis

The descriptive data were expressed by frequencies, means, and standard deviations. All quantitative variables were defined as parametric by the Kolmogorov-Smirnov test. Comparisons of frequencies between groups were performed by the Chi-Square test. The Student's *t*-test was used to compare the means of two parametric sample populations. The Kaplan-Meier method was used for survival analyses and to provide a clear visual representation over time. The Log-Rank test was used for the comparison between curves. Cox proportional hazard regression model was employed for univariate and multivariate (for those variables significant at the univariate analysis) time-dependent analyses, estimating risk by Hazard Ratio (HR) and the respective 95 % Confidence Interval (95 % CI). Cutoff values for continuous variables were established by ROC curve analyses. For all comparisons, a statistical significance level of less than 5 % was adopted (*p* < 0.05), and SPSS version 28.0 (IBM® Inc; Armonk, NY, USA) was used.

## Results

Between 2009 and 2019, 1631 patients were enrolled at the institution under the code ICD-10 C32 (C32, C32.0, C32.1, C32.2, C32.3, C32.8 and C32.9). After applying the inclusion and exclusion criteria, 581 patients were selected for review of neck CT-scans used for pretreatment staging. Imaging review resulted in a total of 321 eligible patients, of which 166 (51.7 %) underwent TL and 155 (48.3 %) received CRT. The participants' demographic characteristics are shown in Table 1. Both groups were also similar between, cT stage, cN stage, age, KPS classification, ECOG classification, and pretreatment BMI.

**Table 1:** Descriptive data and comparisons between both groups of patients, those referred to treatment before and after tumor board implementation.

Variables		Total	Tumor Board		p-value^a^
			Yes	No	
Group	Total Laryngectomy	166	111	55	0.130
		51.7 %	55.0 %	46.2 %	
	Chemoradiation	155	91	64	
		48.3 %	45.0 %	53.8 %	
cT	3	135	76	59	0.039
		42.2 %	37.8 %	49.6 %	
	4a	185	125	60	
		57.8 %	62.2 %	50.4 %	
cN	0	162	107	55	0.592
		50.4 %	53.0 %	46.2 %	
	1	38	21	17	
		11.8 %	10.4 %	14.3 %	
	2a	6	5	1	
		1.9 %	2.5 %	0.8 %	
	2b	54	34	20	
		16.8 %	16.8 %	16.8 %	
	2c	48	27	21	
		15.0 %	13.4 %	17.6 %	
	3b	13	8	5	
		4.0 %	4.0 %	4.2 %	
ECOG classification	0	86	60	26	0.159
		27.7 %	30.5 %	22.8 %	
	1	179	104	75	
		57.6 %	52.8 %	65.8 %	
	2	37	25	12	
		11.9 %	12.7 %	10.5 %	
	3	7	6	1	
		2.3 %	3.0 %	0.9 %	
	4	2	2	0	
		0.6 %	1.0 %	0.0 %	
Age (years)	Number (valid)	317	199	118	0.880
	Mean	60.8	60.8	60.6	
	Standard deviation	9.1	9.1	9.3	
KPS	Number (valid)	310	195	115	0.838
	Mean	85.5	85.6	85.4	
	Standard deviation	10.3	11.2	8.7	
Body Mass Index (kg/m^2^)	Number (valid)	317	200	117	0.691
	Mean	23.3	23.3	23.5	
	Standard deviation	4.9	4.7	5.4	

a p-value obtained by the Chi-Square test for comparisons between groups and Student's *t*-test for the comparison of mean.

At univariate analyses (Table 2), patients submitted to chemoradiation, cN classification, ECOG > 1 and KPS < 80 were the variables associated with the risk of death in patients with LSCC before the implementation of the tumor board. After the intervention, this association was for patients with T4a disease, cN classification, ECOG > 1 and KPS.

**Table 2:** Univariate analysis considering two different periods, before and after tumor board implementation, for death in patients treated for laryngeal squamous cell carcinoma.

Variables	Before tumor board				After tumor board			
	p-value^a^	Hazard ratio	95 % Confidence Interval		p-value^a^	Hazard ratio	95 % Confidence Interval	
			Lower	Upper			Lower	Upper
Chemoradiation (Ref. Total Laryngectomy)	0.003	2.693	1.410	5.145	0.083	0.628	0.371	1.063
T4a stage (Ref. T3)	0.263	0.713	0.395	1.289	0.010	2.184	1.204	3.962
N0	Ref.				Ref.			
N1	0.181	1.747	0.771	3.957	0.076	2.091	0.926	4.726
N2a	0.980	b	b	b	0.965	1.046	0.141	7.783
N2b	0.587	1.280	0.526	3.113	0.063	1.931	0.966	3.859
N2c	0.372	1.434	0.650	3.161	0.000	3.328	1.713	6.467
N3	0.002	7.056	2.015	24.712	0.011	4.028	1.371	11.837
ECOG 0	Ref.				Ref.			
ECOG 1	0.695	0.868	0.428	1.761	0.002	3.144	1.523	6.491
ECOG 2	0.093	2.277	0.873	5.943	0.009	3.455	1.360	8.779
ECOG 3	0.002	38.116	3.723	390.187	0.003	7.143	1.917	26.611
ECOG 4	0.054	1.813	0.991	3.318	0.976	b	b	b
Pretreatment KPS (continuous)	0.104	0.967	0.929	1.007	0.001	0.970	0.952	0.988
Pretreatment IMC (continuous)	0.239	0.966	0.912	1.023	0.591	0.985	0.933	1.040
KPS < 80	0.002	3.868	1.671	8.952	0.173	1.640	0.805	3.342
KPS < 90	0.631	0.859	0.462	1.598	0.000	2.530	1.517	4.220
ECOG > 1	0.009	2.833	1.298	6.183	0.148	1.602	0.846	3.033

Ref., Reference.

a Univariate Cox regression model.

b Reduced number of valid cases reduces the power of the analysis.

The multivariate analyses (Cox regression model) identified that patients submitted to chemoradiation (HR = 2.290; 95 % CI: 1.195‒4.388; *p* = 0.012), with KPS < 80 (HR = 3.420; 95 % CI: 1.478‒7.914; *p* = 0.004), and with N3b disease (HR = 5.228; 95 % CI: 1.463‒18.685; *p* = 0.011) had higher risk of death before the implementation of the tumor board, as demonstrated in Table 3. After the intervention the independent risk factors identified were KPS < 90 (HR = 2.615; 95 % CI: 1.534‒4.460; *p* < 0.001), N2c and N3b disease (respectively HR = 4.293; 95 %CI: 2.171‒8489; *p* < 0.001; and HR = 4.424; 95 % CI: 1.433‒13.656; *p* = 0.010) and chemoradiation was no longer a risk factor for death in LSCC (HR = 0.560; 95 % CI: 0.322‒0.973; *p* = 0.040) as shown in Table 4.

**Table 3:** Multivariate analysis considering the period before tumor board implementation, identifying risk factors of death in patients treated for laryngeal squamous cell carcinoma.

Variables	p-value^a^	Hazard ratio	95 % Confidence Interval	
			Lower	Upper
Chemoradiation (Ref. Total Laryngectomy)	0.012	2.290	1.195	4.388
KPS < 80	0.004	3.420	1.478	7.914
N0	Ref.			
N1	0.498	1.340	0.574	3.127
N2a	0.981	b	b	b
N2b	0.855	1.093	0.421	2.838
N2c	0.302	1.522	0.686	3.379
N3b	0.011	5.228	1.463	18.685

Ref., Reference.

a Multivariate Cox regression model.

b Reduced number of valid cases reduces the power of the analysis. ECOG classification not included in the analysis because it is natural dependent on KPS.

**Table 4:** Multivariate analysis considering the period after tumor board implementation, identifying risk factors of death in patients treated for laryngeal squamous cell carcinoma.

Variables	p-value^a^	Hazard ratio	95 % Confidence Interval	
			Lower	Upper
Chemoradiation (Ref. Total Laryngectomy)	0.040	0.560	0.322	0.973
T4a (Ref. T3)	0.502	1.251	0.651	2.404
KPS < 90	<0.001	2.615	1.534	4.460
N0	Ref.			
N1	0.144	1.856	0.809	4.254
N2a	0.858	0.832	0.111	6.221
N2b	0.061	1.985	0.969	4.068
N2c	<0.001	4.293	2.171	8.489
N3b	0.010	4.424	1.433	13.656

Ref., Reference.

a Multivariate Cox regression model. # Reduced number of valid cases reduces the power of the analysis. ECOG classification not included in the analysis because it is natural dependent on KPS.

**Fig. 1:**
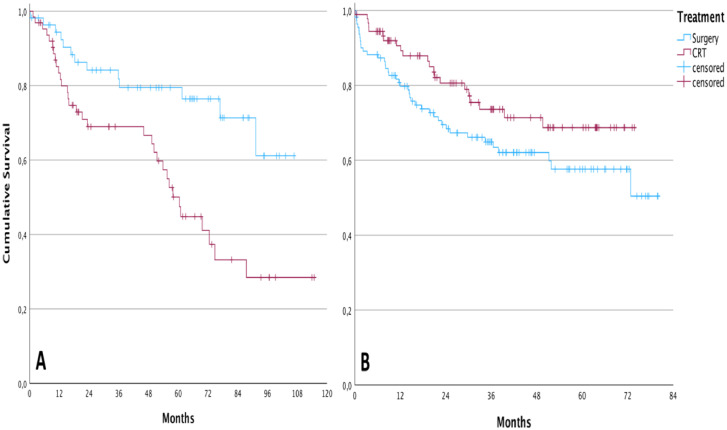
**Improvement of overall survival following tumor board implementation**. (A) Prior to the implementation of the tumor board; (B) After the implementation of the tumor board.

Overall survival in the group of patients undergoing TL was higher than in patients treated with CRT before the implementation of the tumor board (cumulative survival of 61.1 % and 28.4 %, respectively, during the follow-up; *p* = 0.002 – Log-Rank test). After the intervention, the result was the opposite. For patients submitted to chemoradiation, the cumulative overall survival was 68.7 % during the follow-up against 50.4 % for those submitted to total laryngectomy, however, without statistical significance (*p* = 0.081; Log-Rank test). The Kaplan-Meier curves of these analyses are demonstrated in Fig. 1.

## Discussion

The oncological outcomes of patients treated for locally advanced LSCC, according to the treatment modality, were analyzed. The impact of the multidisciplinary discussion on the overall survival of the groups was also studied. Despite being considered the gold standard for the treatment of laryngeal cancer, TL is seen as a mutilating therapy with a huge impact on the patient’s quality of life. TL entails the need for a definitive tracheostomy, difficulty in communication due to the loss of laryngeal voice, social stigma, alteration in the perception of aromas and flavors, and even constipation due to the difficulty in performing the abdominal press^14^^,^^15^. The demand for less morbid therapeutic alternatives culminated in the conduct of several studies aimed at validating new methods^3^^,^^4^. The treatment of laryngeal cancer with CRT, however, should not be understood as free of complications. Laryngeal dysfunction with dysphagia, aspiration, and possible tracheostomy, in addition to xerostomia, hypothyroidism, actinic dermatitis, emesis, and nephrotoxicity, are some of the possible toxicities of CRT.

A historical milestone in the pursuit of new treatments was reported by the Veterans Affairs group in 1991, which showed no inferiority in the survival of advanced LSCC patients treated with neoadjuvant chemotherapy followed by radiotherapy, when compared with a surgically treated group^3^. In the following decades, there was a reduction in the number of TL performed and an increase in the indication of CRT for locally advanced laryngeal tumors^5^^,^^6^. From 1985 to 2014, Lorenzo et al^5^. showed an increase in options for organ-preserving strategies to the detriment of laryngectomies as an initial treatment of patients with locally advanced laryngeal cancer. In that study, patients treated between 2005 and 2014 had worse cancer-specific survival compared to patients treated in the previous decade (1995‒2004)^5^.

A decrease in LSCC patients' survival was observed concurrently with the reduction of TL in the last three decades. Contrary to the trend of increased survival of patients with other head and neck cancers, the larynx subsite is the only one that has not evolved with an improvement in recent decades^16^^,^^17^. It is necessary, however, to respect the complexity involved in the decision by the treatment modality chosen, not only for the consequences already discussed above, but mainly because tumors of different stages respond differently to each type of therapy proposed. Early-stage Tumors (T1‒T2) respond well to endoscopic resection or radiotherapy, with similar overall survival rates^1^. Advanced tumors (T3 and T4a), however, are the subject of extensive discussion in the literature. Forastiere et al^1^. advocated that locally advanced tumors can be treated by organ-preserving surgery, CRT, or isolated radiotherapy; however, extensive T3, larger T4a volume, and/or compromised laryngeal functions evolve with better survival rates and quality of life when submitted to TL. Regarding T3 staged tumors, several studies reported no significant difference in overall survival between those submitted to TL and CRT, unlike T4a, in which the advantage is usually with surgical treatment^5^^,^^6^^,^^11^,^17^, ^18^, ^19^, ^20^, ^21^, ^22^, ^23^, ^24^. In cases of disease recurrence, patient survival and quality of life are better when salvage TL is performed after initial treatment with partial laryngectomy than after CRT^25^.

Timmermans et al^26^. showed no difference in survival rates between patients treated with CRT or TL. However, the authors argued that most T3 patients were treated conservatively, whereas most T4-staged tumors were treated with TL. Reinforcing that there is no consensus in the literatur^17^e on the subject, there are those who defend the non-inferiority of one treatment modality in relation to the other for T3 and T4^27^^,^^28^. More recently, a systematic review published in 2024 concluded that LSCC “T4 tumors should have TL as their treatment of choice” and “in patients with T3 tumors, there are similar survival rates with both treatments” (TL and non-surgical). The study also stated that the chances of recurrence, dysphagia, and feeding tube dependence are greater when using non-surgical therapies^29^.

The present results suggest a slight superiority of surgical treatment compared with the laryngeal conservative treatment. It is possible that this difference might be due to the fact that most of the participants had T4-staged tumors, which, as previously discussed, would benefit more from the surgical modality. When the two studied periods were analyzed separately, however, the difference between the survival of patients submitted to each of the therapeutic modalities was decreased. In fact, CRT treatment was no longer an independent risk factor for lower overall survival, but the opposite. Actually, CRT treatment became an independent protective factor after the intervention. One possible explanation for that result is that multidisciplinary tumor boards caused better patient selection for each therapy modality, raising the outcome of both groups: TL and CRT.

The management of locally advanced LSCC is challenging and requires assessment by an experienced multidisciplinary team. Patient expectations, tumor extension, pre-treatment laryngeal function, and the existence of comorbidities are critical factors for selecting surgical or non-operative treatments^12^. The Charlson-Deyo comorbidity index, for example, can influence the choice of therapeutic modality^11^. Socio-demographic factors also influence the choice of treatment for patients with advanced LSCC. A population-based study of 5381 patients treated between 1992 and 2009 showed that younger (< 60-years) and poorer patients were more likely to receive surgical treatment; women and married people had a survival advantage, and afro-descendants had a worse prognosis^13^. More advanced T and N staging require more time for discussion in multidisciplinary meetings^30^.

Timme et al^31^. published the hypothesis that multidisciplinary treatment planning optimized the indication of organ preservation protocols in LSCC, resulting in no compromised survival in patients with T3 or T4a staged tumors compared with those operated on.

A meta-analysis published by Oxford University Press in 2024 “found a significant positive effect of multidisciplinary cancer conferences compared with controls”. That study analyzed 59 publications, with 134,287 patients, and concluded that those meetings increased overall survival^32^.

Since 2014, the discussion of advanced LSCC cases has become a regular topic during institution head and neck tumor board meetings. The approximation of the survival curves of operated and non-operated patients, after 2014, suggests that multidisciplinary meetings were beneficial to patients, as they better selected a treatment modality for each case. Indeed, CRT was no longer considered as a risk factor to poorer survival after the tumor board implementation, which could mean that non-surgical treatment was more judiciously indicated. Of note, no other relevant alterations were identified in the care provided to patients with LSCC at ICESP from that year onwards (including chemotherapy regimen, radiation doses, radiation fields and surgical techniques).

In a multidisciplinary discussion, the participation of the patient is essential. The patient's opinion, values, and environment are essential for the multidisciplinary team to decide on therapeutic planning, especially in cases where surgical and non-surgical treatments offer the same survival outcome, as in T3 and low-volume T4a. In Brazil, a developing country with marked social disparity, it is worth considering, for example, whether the patient is literate when discussing the best therapy, because losing the ability to speak can result in social and economic exile. Even phonatory rehabilitation techniques (whether with esophageal voice, tracheoesophageal prosthesis, or electronic larynx) can be too challenging for those with very low levels of education^33^. Patients with head and neck cancer more frequently refuse proposals for surgical treatment when the tumors are of a more advanced stage and with cancer of the laryngeal and hypopharyngeal subsites^34^. Educational status, however, was not found to be a predictive factor for refusing recommended medical treatment in the US population^35^.

## Conclusion

Finally, the treatment of locally advanced LSCC can be performed with TL or CRT, with TL being preferred for patients with T4a stage tumors. A multidisciplinary discussion among professionals with experience in caring for patients with head and neck cancer has a positive impact on the oncological outcomes. The therapeutic decisions taken in tumor boards is highly recommended.

## Ethics approval statement

The study was also approved by the Institutional’s Review Board (CAAE: 38,792,420.1.0000.0068).

## Authors’ contributions

Mariana Gonçalves Rodrigues: Conceptualization; Data curation; Investigation; Project administration; Validation; Roles/Writing.

Daniel Marin Ramos: Conceptualization; Data curation; Investigation; Methodology; Project administration; Supervision; Validation.

Leandro Luongo de Matos: Formal analysis; Validation.

Chin Shien Lin: Formal analysis; Validation.

Jorge Tomio Takahashi: Data curation; Investigation; Validation

Tiago Wanderley Diniz Chamel: Data curation; Investigation; Validation.

Luiz Paulo Kowalski: Conceptualization; Methodology; Project administration; Supervision; Validation.

## Funding

This research did not receive any specific grant from funding agencies in the public, commercial, or not-for-profit sectors.

## Data availability statement

The datasets generated and/or analyzed during the current study are available from the corresponding author upon reasonable request.

## Declaration of competing interest

The authors declare no conflicts of interest.
